# Kinase-independent inhibition of cyclophosphamide-induced pathways protects the ovarian reserve and prolongs fertility

**DOI:** 10.1038/s41419-019-1961-y

**Published:** 2019-09-27

**Authors:** Giovanna Bellusci, Luca Mattiello, Valentina Iannizzotto, Sarah Ciccone, Emiliano Maiani, Valentina Villani, Marc Diederich, Stefania Gonfloni

**Affiliations:** 1 Department of Biology, University of Rome Tor Vergata, via della Ricerca Scientifica, 00133 Rome, Italy; 2 College of Pharmacy, Seoul National University, 1 Gwanak-ro, Gwanak-gu, Seoul, 08826 Republic of Korea

**Keywords:** Apoptosis, Drug development

## Abstract

Premature ovarian failure and infertility are adverse effects of cancer therapies. The mechanism underlying chemotherapy-mediated depletion of the ovarian reserve remains unclear. Here, we aim to identify the signaling pathways involved in the loss of the ovarian reserve to prevent the damaging effects of chemotherapy. We evaluated the effects of cyclophosphamide, one of the most damaging chemotherapeutic drugs, against follicle reserve. In vivo studies showed that the cyclophosphamide-induced loss of ovarian reserve occurred through a sequential mechanism. Cyclophosphamide exposure induced the activation of both DNAPK-γH2AX-checkpoint kinase 2 (CHK2)-p53/TAp63α isoform and protein kinase B (AKT)-forkhead box O3 (FOXO3a) signaling axes in the nucleus of oocytes. Concomitant administration of an allosteric ABL inhibitor and cyclophosphamide modulated both pathways while protecting the ovarian reserve from chemotherapy assaults. As a consequence, the fertility of the treated mice was prolonged. On the contrary, the administration of an allosteric ABL activator enhanced the lethal effects of cyclophosphamide while shortening mouse fertility. Therefore, kinase-independent inhibition may serve as an effective ovarian-protective strategy in women under chemotherapy.

## Introduction

Premature ovarian failure and infertility are frequent consequences of cancer therapy^1^. Chemotherapy triggers the degeneration of the ovarian reserve^2,3^, a source of fertilizable eggs during the entire reproductive life of a female. Preserving the ovarian reserve during cancer treatment is a major concern for the maintenance of fertility and to ameliorate the quality of life of survivors. Studies in transgenic mouse models have revealed the role of several molecules in the maintenance of ovarian reserve following ionization radiation (IR) treatment. The alpha TAp63 isoform (TAp63α) was thought to be a key mediator of IR-induced ovarian reserve loss^4^. Lack of TAp63α expression in mouse oocytes promoted resistance to the lethal effects of IR^5^. IR-induced TAp63 activation depended on a conformational transition of the molecule^6^. The pro-apoptotic proteins PUMA and NOXA may act as downstream effectors of TAp63α^7^. In mice, checkpoint kinase 2 (CHK2) is essential for DNA damage surveillance in female meiosis^8^. Moreover, the lack of CHK2 expression affects TAp63 activation following IR treatment^8^. In vitro experiments in ovarian culture system showed that the transient administration of a CHK2 inhibitor II hydrate may preserve oocyte viability following IR treatment^9^. Another study demonstrated that CHK2 inhibitor II hydrate affected the activities of a broad spectrum of kinases and impacted the global repression of the DNA damage response (DDR) in cultured ovaries^10^, thereby raising some questions about its specificity. Tuppi et al. reported that CHK2 and the executioner kinase CK1 are both involved in the TAp63α-mediated oocyte degeneration in cultured ovaries exposed to chemotherapy^11^. As cultured mouse ovaries exposed to chemotherapeutic drugs may not recapitulate the signaling pathways physiologically occurring in the ovary in vivo, we aimed to perform all our experiments under in vivo conditions. We evaluated the effects of cyclophosphamide, a chemotherapeutic agent commonly used for the treatment of pediatric cancer patients. Cyclophosphamide is transformed in the liver into active alkylating metabolites, which induce the formation of DNA adducts. How cyclophosphamide contributes to ovarian follicle depletion is yet incompletely understood and is debatable^12^. Meirow et al. suggest that the mechanism underlying cyclophosphamide-induced oocyte loss comprised the accelerated activation of primordial follicle that results in a “burnout” effect^13^. However, chemotherapeutic drugs may directly damage the primordial follicle and induce apoptosis. The extent of ovarian reserve loss also depends on the type and dosage of the chemotherapeutic agent^14^. Understanding the molecular basis underlying the effect of chemotherapy on quiescent primordial follicles is therefore essential for the identification of an effective co-treatment that may simultaneously preserve fertility in women^15,16^. Here, we investigated the in vivo consequences of different doses of cyclophosphamide on the ovarian reserve. We used a pre-pubertal mouse model to evaluate the effect of transient administration of small molecule kinase inhibitors to identify putative ferto-protective adjuvants^17^ to limit the damaging effects of chemotherapy.

## Results

### Cyclophosphamide triggers a dose-dependent loss of primordial follicles

**Fig. 1:**
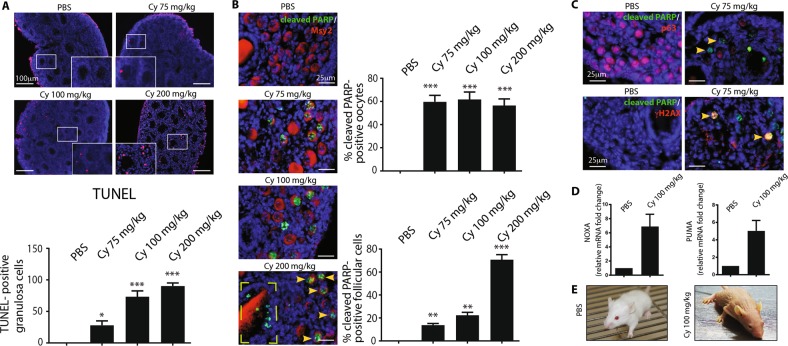
Cyclophosphamide triggers apoptosis in pre-pubertal mouse ovary in a dose-dependent manner. P7 mice were injected with vehicle (PBS) or increasing concentrations of cyclophosphamide (75, 100, and 200 mg/kg) and sacrificed within 16 h from injection. Ovarian sections were analyzed by in situ TdT-mediated dUTP nick-end labelling (TUNEL) assay. **a** A small area of each image was selected and zoomed in (magnification box). The graph shows the quantification of TUNEL-positive cells. Quantification of TUNEL-positive cells was performed by counting six different middle ovarian sections derived from three distinct ovaries. Follicle apoptosis was assessed by IF assay with two specific antibodies against cleaved PARP (green) and Msy2 (red), a cytoplasmic marker of germ cells. **b** Yellow arrows at the bottom indicate positive oocytes, while the dashed box illustrates PARP positive-granulosa cells from large growing follicle. Quantification of cleaved PARP-positive cells was performed by counting several (6 < *x* < 10) middle ovarian sections derived from three distinct ovaries. Scale bar magnification, 100 μm for TUNEL assay and 25 μm for IF assay. **c** Follicle apoptosis was assessed by IF assay with two specific antibodies against cleaved PARP (green) and p63 (red), a nuclear marker of germ cells. We also monitored γH2AX, a marker of DDR, in the reserve oocytes (yellow arrows). **d** The pro-apoptotic PUMA and NOXA genes expression was assessed by RT-qPCR; GAPDH was used as reference control; data are shown as fold change vs PBS. **e** Mouse images were obtained 7 days after cyclophosphamide injection. **a**, **b**, **d** Bar column represents mean ± s.d.; statistical significance was determined using one-way analysis of variance (ANOVA) (**P < 0.01; ***P < 0.001 as compared with PBS-treated group)

We investigated the effects of cyclophosphamide on perinatal mouse ovaries. We injected mice (at postnatal day 7, P7) with different doses of cyclophosphamide (ranging from 50 to 200 mg/kg). One day after injection, ovaries were dissected and analyzed with TdT-mediated dUTP nick-end labelling (TUNEL), immunofluorescence (IF), and immunohistochemistry (IHC) assays. Cyclophosphamide administration induced a dose-dependent increase in the number of TUNEL-positive granulosa cells surrounding the growing follicles (Fig. 1a). Cell death in the ovarian follicle reserve was observed with IF staining for cleaved poly ADP ribose polymerase (PARP) (Fig. 1b). We found apoptotic oocytes in the groups treated with low doses of cyclophosphamide (50–75 mg/kg), while higher doses of cyclophosphamide (100–200 mg/kg) activated cleaved PARP in the granulosa cells of large growing follicles (see dashed yellow box in Fig. 1b). We also detected the phosphorylation of the histone variant H2AX at Ser139 residue, also named as γH2AX, an early hallmark of DDR, in the nucleus of cyclophosphamide-damaged oocytes (Fig. 1c). We also detected the enhanced transcription of PUMA and NOXA genes by RT-qPCR (Fig. 1d). Temporary hair loss was reported a week later in cyclophosphamide-injected animals (Fig. 1e).

### DNAPK is activated in the nucleus of reserve oocytes following cyclophosphamide treatment

**Fig. 2:**
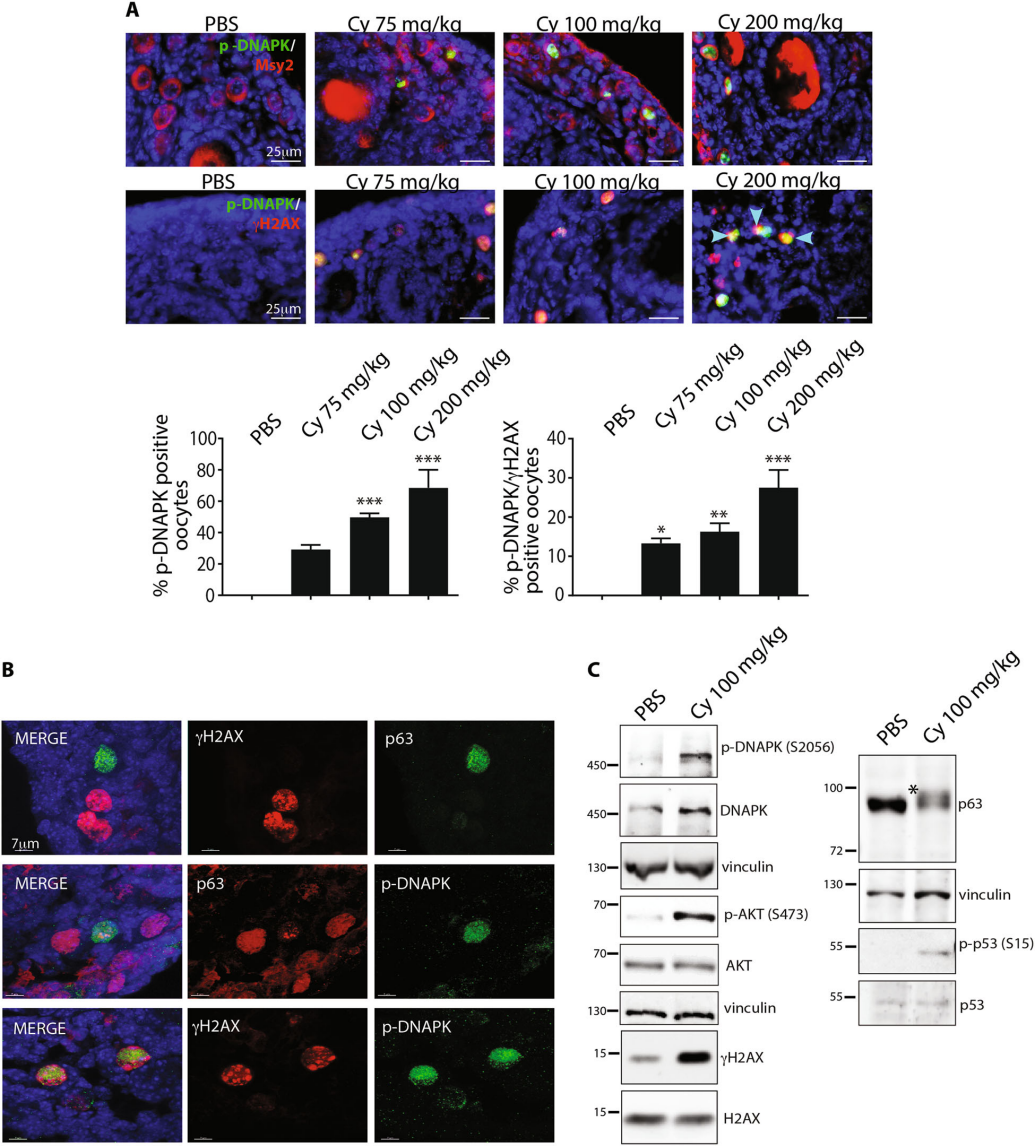
DNA-PK is activated in the nucleus of reserve oocytes following cyclophosphamide treatment. P7 mice were injected with vehicle (PBS) or increasing concentrations of cyclophosphamide (75, 100, and 200 mg/kg) and sacrificed within 16 h after injection. DNAPK activation was evaluated with IF assay using a phospho-specific antibody (green) and Msy2 (red), a cytoplasmic antigen of germ cells (**a**). Blue arrows at the bottom indicate the oocytes positive for p-DNAPK and γH2AX. Quantification was performed by counting several (6 < *x* < 8) middle ovarian sections derived from distinct ovaries. **b** Confocal images of middle ovarian sections from cyclophosphamide-injected mice indicated that the oocytes with high expression of p-DNAPK or γH2AX showed reduced expression of TAp63. **c** Western blot analysis of ovarian lysates for female pups injected with 100 mg/kg of cyclophosphamide or PBS. Cyclophosphamide treatment induced a mobility shift of TAp63 (indicated with an asterisk [*]) that was absent in the control. Scale bar magnification, 25 μm for IF assay and 7 μm for confocal images. **a** Bar column represents mean ± s.d.; statistical significance was determined using one-way analysis of variance (ANOVA) (*P < 0.5; **P < 0.01; ***P < 0.001 as compared with PBS-treated group)

To investigate the mechanism underlying cyclophosphamide-induced follicle death, we evaluated the activation of the apical DDR kinase (DNAPK) with phospho-specific antibodies after 16 h of cyclophosphamide injection. The presence of apoptosis in the primordial/primary follicle population was consistent with the activation of DNAPK in the nucleus of reserve oocytes (Fig. 2a). Phosphorylation of the histone variant H2AX at Ser139 was observed in the nucleus of damaged oocytes. The majority of oocytes were positive for both DNAPK and γH2AX expression at every dose of cyclophosphamide (Fig. 2a). Confocal images of middle ovarian sections obtained from cyclophosphamide-injected mice showed that the oocytes with a strong signal for γH2AX or p-DNAPK had lower expression of TAp63 (Fig. 2b). Western blot analysis of the dissected ovaries revealed the cyclophosphamide-induced phosphorylation of both γH2AX and p53. In addition, cyclophosphamide-induced TAp63 activation, as indicated by a mobility shift (*) that was absent in the control group (Fig. 2c).

We observed activation of checkpoint kinase CHK2 and p53 in the nucleus of the damaged oocytes (Supplementary Fig. 1A). Both CHK2 and p53 are involved in the removal of oocytes with unrepaired meiotic DNA double-strand breaks (DBSs)^8^. Confocal images show phosphorylated p53 and concomitant lower expression of TAp63 in the oocyte nucleus (Supplementary Fig. 1B). ChIP assay performed on cyclophosphamide-injected ovaries shows that p53 activation led to transcription of proapoptotic gene PUMA (Supplementary Fig. 1C). Thus, the cyclophosphamide-induced loss of ovarian reserve involves the activation of the DNAPK-γH2AX-CHK2-p53/TAp63α signaling axes.

We investigated the mechanisms underlying the cyclophosphamide-induced follicle death following the activation of the protein kinase B (AKT) pathway using phospho-specific antibodies after 16 h treatment with cyclophosphamide (Supplementary Fig. 2). The AKT pathway is involved in primordial follicle activation through the regulation of the activity of the forkhead box O3 (FOXO3a) transcription factor^18^. Upon phosphorylation, AKT may enter the nucleus and phosphorylate FOXO3a, which eventually leaves the nucleus and enables follicular activation.

Following cyclophosphamide injection, p-AKT and p-FOXO3a were detected by confocal microscopy (Supplementary Fig. 2B) in the nucleus of reserve oocytes and in a dose-dependent manner (Supplementary Fig 2A, upper and lower panel). We also observed concomitant expression of p-AKT and γH2AX (Supplementary Fig. 2A, central panel) and p-AKT/pFOXO3a with p-ATM (Supplementary Fig. 2C). Thus, the presence of the early marker of DDR after cyclophosphamide treatment was consistent with the activation of the AKT-FOXO3a pathway.

### GNF-2 prevents γH2AX phosphorylation in the nucleus of reserve oocytes

Previous studies have revealed the protective role of an ABL kinase inhibitor (imatinib) on ovarian reserve in cisplatin-induced degeneration^19–22^. However, the mechanism underlying oocyte protection by imatinib remains unclear. As discussed in a recent paper^10^, cisplatin induced a response that activated the pathways different from those activated by IR. Cisplatin toxicity gradually accumulated from multiple sources (lipids, proteins, etc.) and induced integrated pathways of the two p53 homologs (TAp63α and TAp73α)^10^ in follicles.

**Fig. 3:**
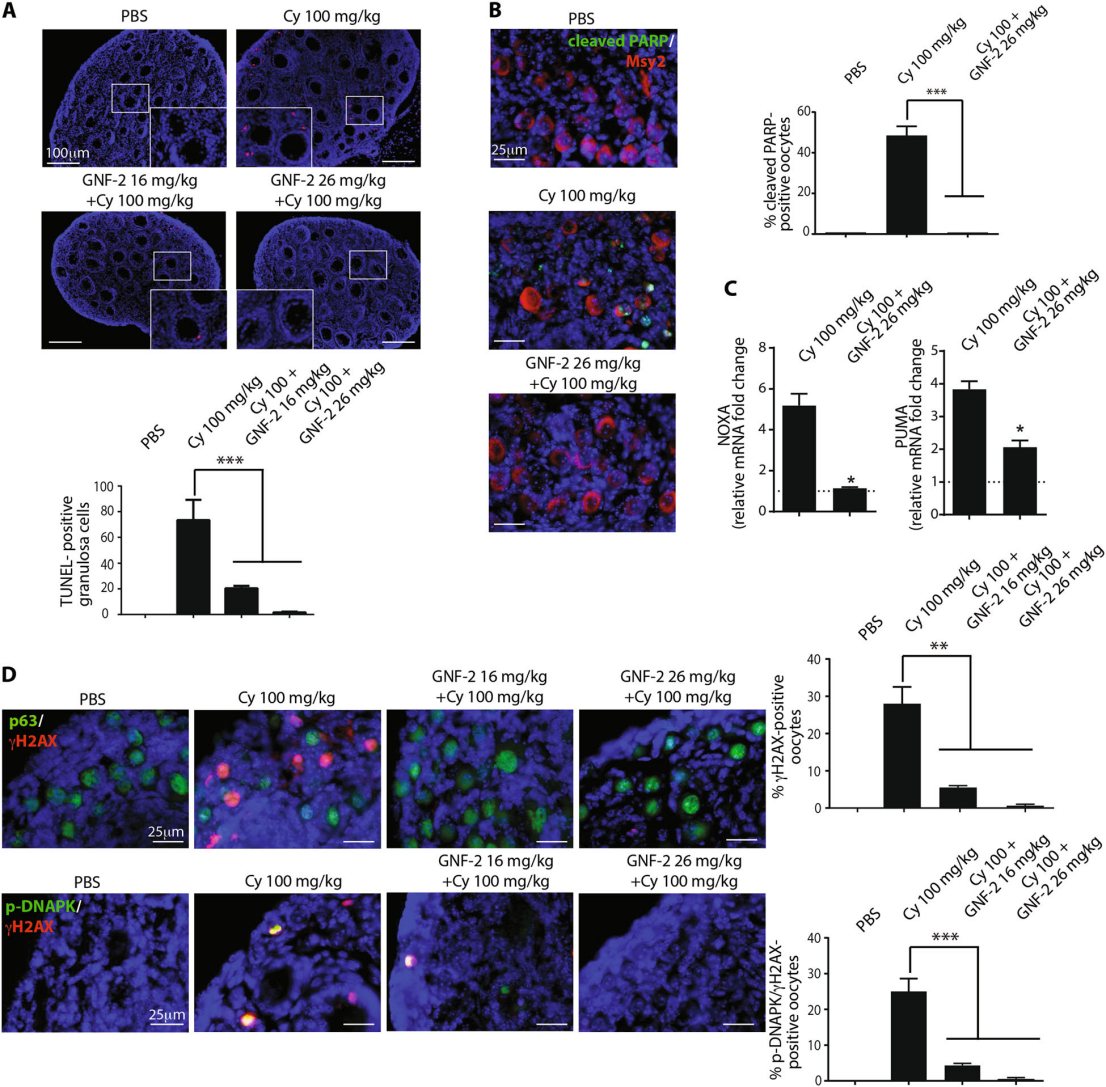
GNF-2 prevents the cyclophosphamide-induced DNAPK-γH2AX signaling. P7 mice were injected with vehicle (PBS) or cyclophosphamide (100 mg/kg) with/without increasing concentrations of GNF-2 (16 and 26 mg/kg). Mice were sacrificed within 16 h after injection. **a** Ovarian sections were analyzed by in situ TdT-mediated dUTP nick-end labelling (TUNEL) assay. The graph shows the quantification of TUNEL-positive cells. Quantification of TUNEL-positive cells was performed by counting six different middle ovarian sections derived from three distinct ovaries. **b** Follicle reserve apoptosis was assessed by IF assay with two specific antibodies against cleaved PARP (green) and Msy2 (red), a cytoplasmic antigen of germ cells. Quantification of the cleaved PARP-positive cells was performed by counting several (6 < *x* < 8) middle ovarian sections derived from three distinct ovaries. **c** The pro-apoptotic PUMA and NOXA gene expression was assessed by RT-qPCR; GAPDH was used as reference control; Data are shown as fold change vs PBS represented by a dashed line. **d** γH2AX and DNAPK activation was investigated with IF assay using phospho-specific antibodies (red) and anti-p63 (green), a nuclear marker for germ cells. Quantification was performed by counting several (6 < *x* < 8) middle ovarian sections derived from three distinct ovaries. Scale bar magnification, 100 μm for TUNEL assay and 25 μm for IF assay. **a**, **b**, **d** Bar column represents mean ± s.d.; statistical significance was determined using one-way analysis of variance (ANOVA) (**P < 0.01; ***P < 0.001 as compared to 100 mg/kg cyclophosphamide). **c** Bar column represents mean ± s.e.m.). Statistical significance was determined by unpaired Student’s *t* test (*P < 0.05 compared to Cy 100 mg/kg)

Here, we tested whether the allosteric ABL compounds affect the DDR induced by cyclophosphamide. In our experiments, we used an allosteric activator of ABL kinase^23^ (Supplementary Fig. 3) to validate the effect of ABL compounds in vivo. We treated female mice with cyclophosphamide in the presence of GNF-2. Concomitant administration of GNF-2 resulted in a significant reduction in the number of TUNEL-positive granulosa cells in a dose-dependent manner (Fig. 3a). We failed to observe any apoptosis in reserve oocytes (Fig. 3b). Co-treatment of cyclophosphamide and GNF-2 also affected DNAPK activation and the consequent γH2AX phosphorylation in the nucleus of reserve oocytes (Fig. 3d). Also, the transcription of proapoptotic genes PUMA and NOXA is affected by GNF-2 (Fig. 3c).

**Fig. 4:**
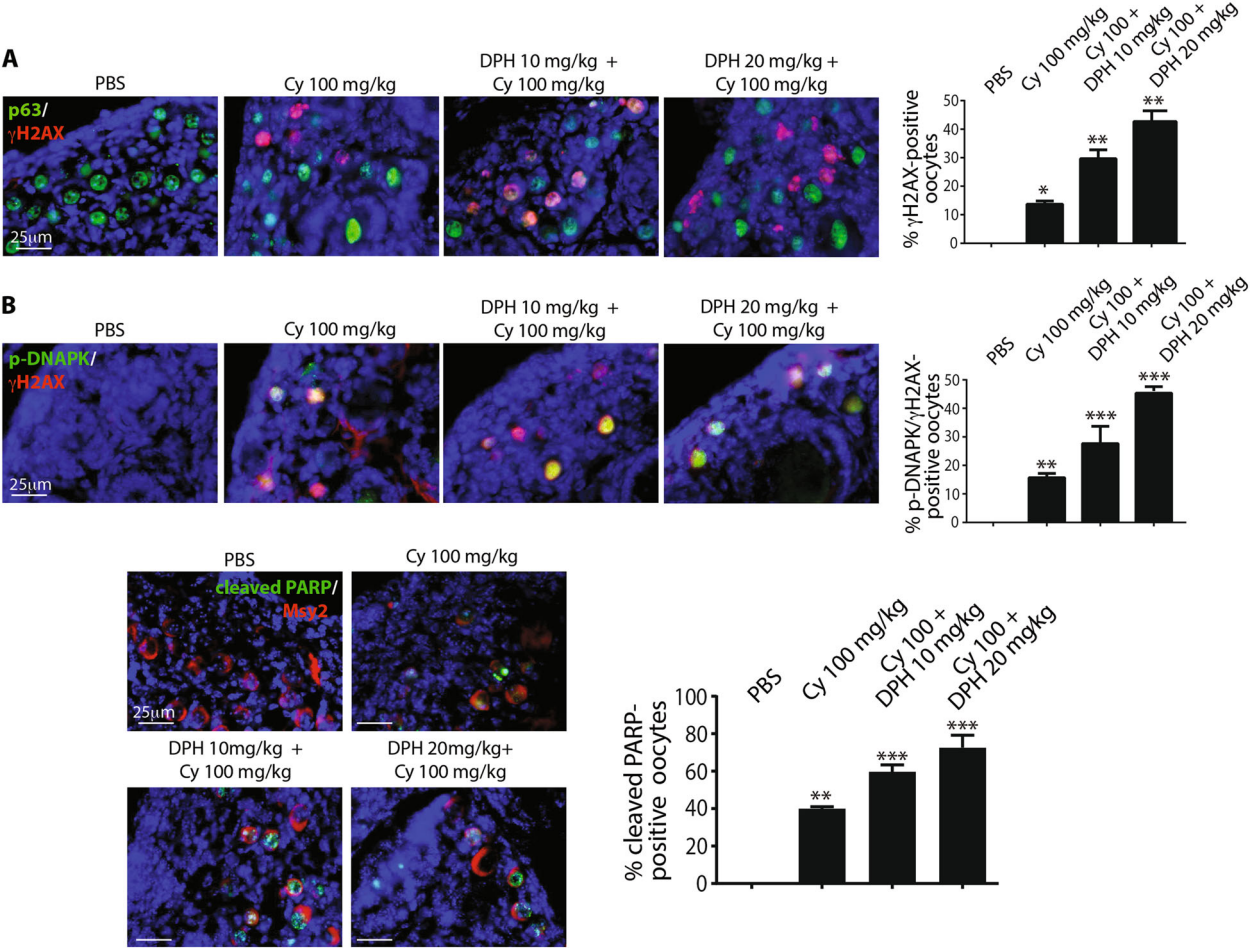
DPH enhances the DNAPK-γH2AX signaling axis and oocyte apoptosis induced by cyclophosphamide. P7 mice were injected with vehicle (PBS) or cyclophosphamide (100 mg/kg) with/without increasing concentrations of DPH (10 and 20 mg/kg) and were sacrificed within 16 h from injection. **a**, **b** γH2AX and DNA-PK activation was evaluated with IF assay using phospho-specific antibodies (red), while p63 (green) was used as a nuclear marker for germ cells. Follicle reserve apoptosis was assessed by IF assay with two specific antibodies against cleaved PARP (green) and Msy2 (red), a cytoplasmic marker of germ cells. Quantification of cleaved PARP-positive cells was performed by counting several (6 < *x* < 8) middle ovarian sections derived from three distinct ovaries. Quantification was performed by counting several (6 < *x* < 8) middle ovarian sections derived from three distinct ovaries. Scale Bar magnification, 25 μm. Bar column represents mean ± s.d.; statistical significance was determined using one-way analysis of variance (ANOVA) (*P < 0.05; **P < 0.01; ***P < 0.001 as compared with PBS-treated group)

On the contrary, the concomitant administration of DPH (an allosteric ABL activator) enhanced DDR activation (Fig. 4) and oocyte apoptosis induced by cyclophosphamide.

IF assay results revealed the increase in the number of γH2AX-positive oocytes (Fig. 4a), p-DNAPK positive oocytes (Fig. 4b) and cleaved PARP positive oocytes (Fig. 4c) after cyclophosphamide treatment. Therefore, we investigated whether ATM phosphorylates H2AX in the damaged oocytes. IF assay results showed that ATM phosphorylation was coupled with a decrease in TAp63 expression in the nucleus of reserve oocytes, as reported for p-DNAPK (Supplementary Fig. 4). The concomitant administration of GNF-2 also prevented ATM activation in the nucleus of reserve oocytes.

In conclusion, GNF-2 and cyclophosphamide co-treatment restricted apoptosis in ovarian reserve through the prevention of the activation of the DNAPK(ATM)-γH2AX-CHK2-p53/Tap63 signaling axes. We also evaluated the effect of allosteric compounds on the activation of the AKT-FOXO3a pathway. Co-treatment of cyclophosphamide with GNF-2 prevented the translocation of p-AKT in the nucleus of the oocyte (Supplementary Fig. 5A), while DPH enhanced the activation of AKT and concomitant phosphorylation of FOXO3a (Supplementary Fig. 5D, E) in the ovarian reserve assaulted by cyclophosphamide.

### GNF-2 prevents the cyclophosphamide-induced loss of follicle reserve

**Fig. 5:**
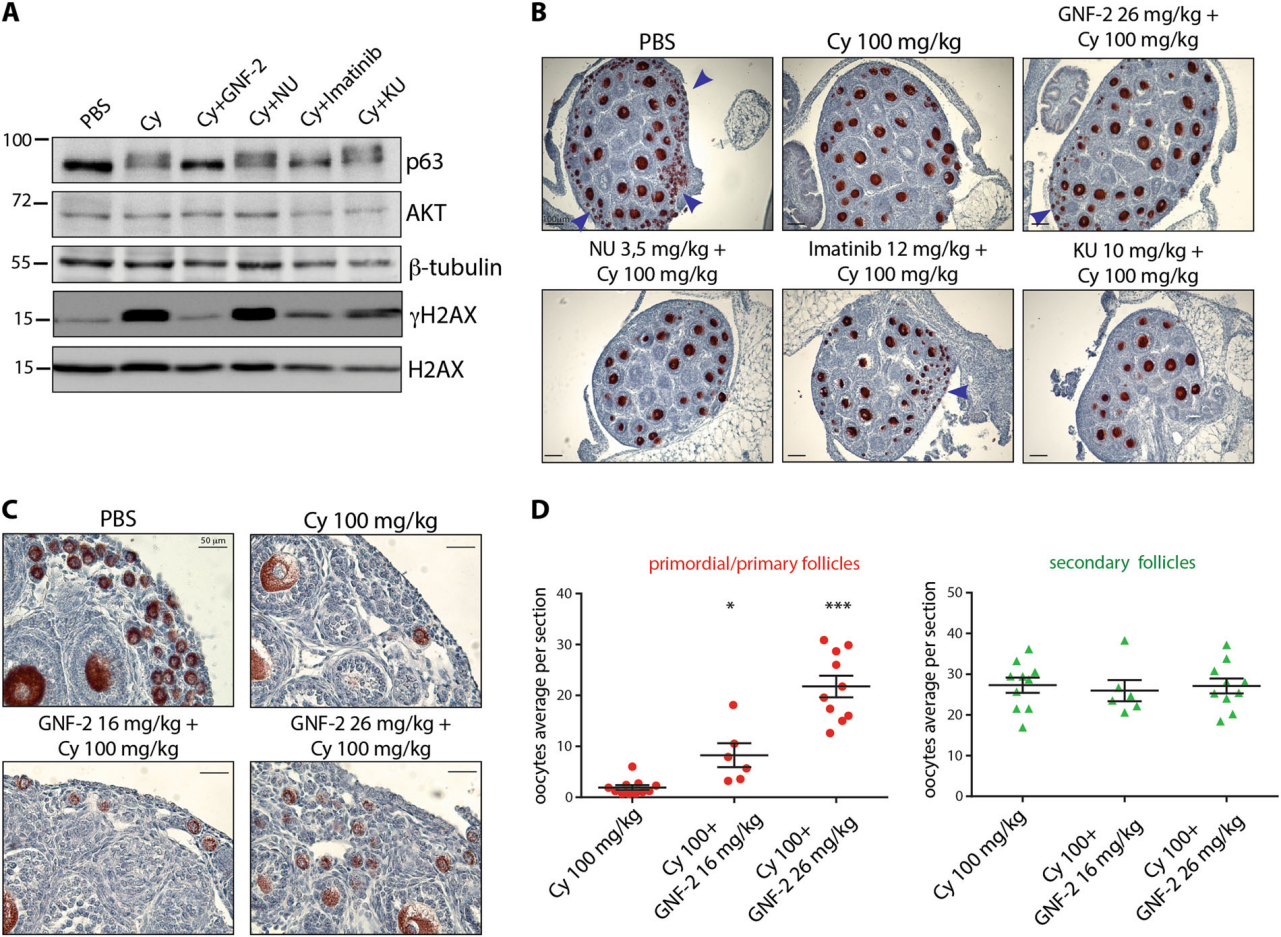
GNF-2 prevents cyclophosphamide-induced loss of reserve follicles. P7 mice were injected with vehicle (PBS) or cyclophosphamide (100 mg/kg) alone or in combination with different kinase inhibitors (GNF-2, imatinib, NU7441, or KU55933). Mice were sacrificed within 24 h from injection. **a** Western blot analysis showed that GNF-2 prevented TAp63 activation/shift and γH2AX phosphorylation, whereas NU7441 and KU55933 were less effective in preventing TAp63 activation/shift. **b**, **c** Ovaries were dissected 3 days after injection and analyzed with IHC assay using Msy-2 antibody. **d** Several ovaries from three independent experiments were analyzed; each dot in the box plot represents the average number (primordial/primary or secondary) of follicles per section of each gonad collected. Scale Bar magnification, 100 μm (**b**), 50 μm (**c**). Statistical significance was determined using one-way analysis of variance (ANOVA) (*P < 0.05; ***P < 0.001 as compared with the group treated with 100 mg/kg cyclophosphamide). Blue arrows indicate follicle reserves

We dissected and analyzed the cyclophosphamide-treated ovaries by western blotting. Ovarian lysates from mice injected with PBS or cyclophosphamide (100 mg/kg) alone or in combination with kinase inhibitors targeting ABL (GNF-2, imatinib), DNAPK (NU7441), or ATM (KU55933) revealed the GNF-2-mediated prevention of TAp63 activation (i.e., absence of mobility shift) and γH2AX generation. NU7441 and KU55933 did not prevent TAp63 activation or γH2AX generation (Fig. 5a). On the other hand, imatinib partially prevented TAp63 activation/shift and γH2AX expression. These results were also confirmed by IHC assay performed on the ovaries dissected 3 days after injection (Fig. 5b). Ovarian sections showed a massive depletion in primordial and primary follicles after cyclophosphamide treatment, whereas a significant rescue in follicle reserve was observed in the ovaries co-treated with cyclophosphamide and GNF-2 (Fig. 5c). We counted primordial, primary, and secondary follicles from middle-ovary sections (Fig. 5d), as previously described^22^. We failed to observe any significant difference in secondary follicles in cyclophosphamide-treated and control groups.

### Imatinib and NU7441 partially mitigate the toxic effect of cyclophosphamide

**Fig. 6:**
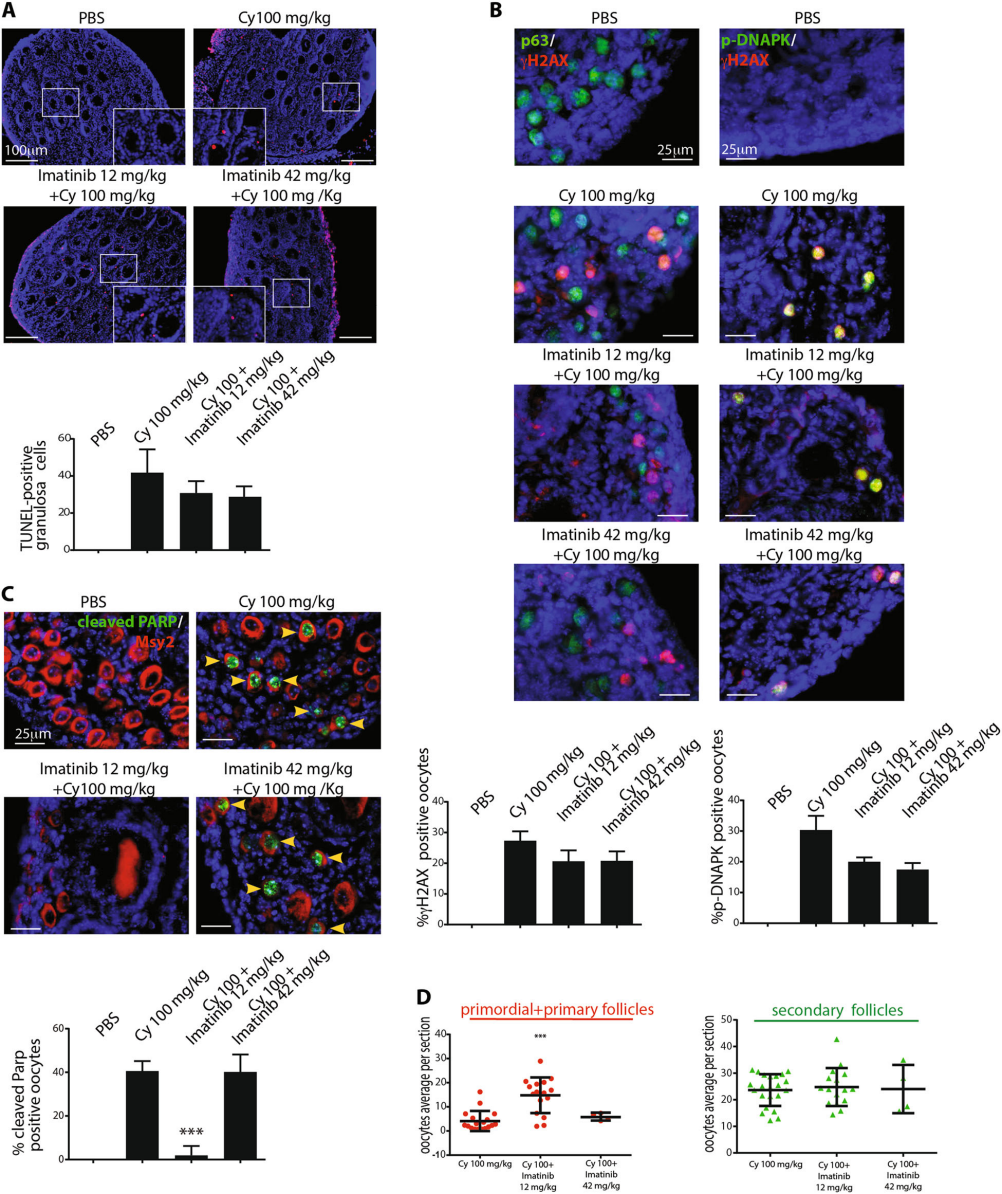
Imatinib partially prevents oocyte apoptosis induced by cyclophosphamide. P7 mice were injected with vehicle (PBS) or cyclophosphamide (100 mg/kg) with/without increasing concentrations of imatinib (12 and 42 mg/kg) and sacrificed within 16 h from injection. **a** Ovarian sections were analyzed by in situ TdT-mediated dUTP nick-end labelling (TUNEL) assay. The graph shows the quantification of TUNEL-positive cells. Quantification of TUNEL-positive cells was performed by counting six different middle ovarian sections derived from three distinct ovaries. **b** γH2AX and DNAPK activation was observed with IF assay using phospho-specific antibodies and p63 was used as a nuclear marker for germ cells. Quantification was performed by counting several (6 < *x* < 8) middle ovarian sections derived from three distinct ovaries. Co-staining for p-DNAPK and γH2AX showed the activation of DNA damage response in reserve oocytes. Quantification was performed by counting several (6 < *x* < 8) middle ovarian sections derived from three distinct ovaries. **c** Ovarian reserve apoptosis was assessed by IF assay using antibodies against cleaved PARP (green) and Msy2 (red), a cytoplasmic antigen of germ cells. Quantification of cleaved PARP-positive cells was performed by counting several (6 < *x* < 8) middle ovarian sections derived from three distinct ovaries. **a**–**c** Bar column represents mean ± s.d.; statistical significance was determined using one-way analysis of variance (ANOVA) (***P < 0.001 as compared with the group treated with 100 mg/kg cyclophosphamide). **d** Ovaries dissected 3 days after injection were analyzed with IHC assay using Msy2 antibody (Fig. 5b). Ovaries from three independent experiments were analyzed; each dot in the box plot represents the average number of follicles (primordial + primary and secondary) per section of each gonad collected. Statistical significance was determined using one-way analysis of variance (ANOVA) (***P < 0.001 as compared with cyclophosphamide-treated group at 100 mg/kg). Scale bar magnification, 100 μm for TUNEL assay and 25 μm for IF assay

We evaluated the effect of different doses of two kinase inhibitors imatinib and NU7441. Both compounds bind to the ATP-binding cleft of kinases and are quite selective for their main target kinases. However, imatinib binds to other kinases, such as platelet-derived growth factor receptor (PDGFR) and c-KIT^24^, while NU7441 also inhibits phosphatidylinositol-4,5-bisphosphate 3-kinase (PI3K) and has little activity against ATM and ATR^25^. We assessed the effect of these compounds in combination with cyclophosphamide. We injected mice with cyclophosphamide (100 mg/kg) in the presence of each inhibitor. TUNEL assay result showed that the co-treatment with imatinib or NU7441 had milder effects on the prevention of granulosa cell death than co-treatment with GNF-2 (Fig. 6a and Supplementary Fig. 6A). We found that imatinib and NU7441 failed to prevent DNAPK-γH2AX-TAp63 activation (Fig. 6b and Supplementary Fig. 6B). However, at low dose, imatinib temporarily prevented apoptosis in follicle reserve (Fig. 6c). This observation was also confirmed by IHC assay (Fig. 5b) performed in ovarian sections dissected after 3 days of injection (Fig. 6d). NU7441 and cyclophosphamide co-treatment mildly mitigated apoptosis in follicle reserve (Supplementary Fig. 6C) as confirmed by IHC staining (Fig. 5b) of the ovarian sections dissected 3 days after injection (Supplementary Fig. 6D). In conclusion, imatinib and NU7441 are less effective than the allosteric inhibitor GNF-2 in the prevention of cyclophosphamide-induced follicle loss. This observation may depend on the inhibition of kinases, including the receptor tyrosine kinase c-KIT and PIK3 that play important roles in follicle reserve. On the contrary, no known target for the allosteric compound GNF-2, other than ABL kinases, has been reported so far^26^.

### GNF-2 prolongs fertility in female mice injected with cyclophosphamide

**Fig. 7:**
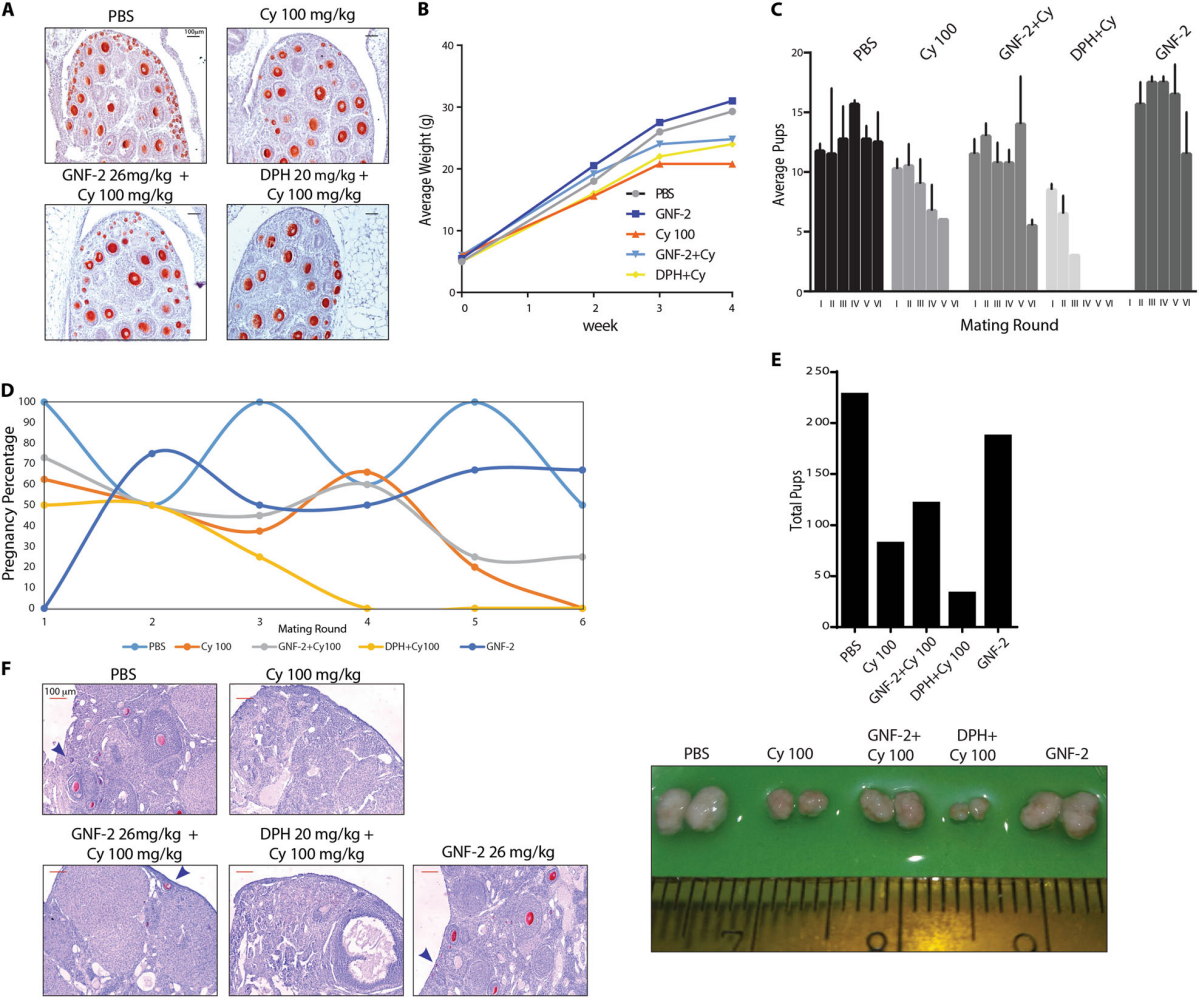
Co-treatment with allosteric compounds modulates fertility in cyclophosphamide-injected mice. **a** Ovaries of each experimental group were dissected 3 days after injection and analyzed with IHC assay using Msy-2 antibody. **b** Graph of average weight following 4 weeks from injection for each experimental group. **c** Time course change in litter size of each group. **d** Percentage of pregnancy rate for the experimental groups throughout mating rounds. **e** Total number of pups delivered by females of each experimental group. **f** Gross morphologies and representative sections of the same ovaries analyzed by IHC assay with Msy2 antibody. Scale bar, 100 μm

To investigate the long-term effect of concomitant GNF-2 administration, co-treated mice were allowed to grow and eventually mated with fertile males. Mice were injected at P7, 3 days after few ovaries from each experimental group were analyzed with IHC assay (Fig. 7a and Supplementary Fig. 7A). We also monitored hair recovery for each experimental group after 2 weeks of injection (Supplementary Fig. 7B) and evaluated pubertal ovaries of cyclophosphamide-treated mice by IHC staining before fertility test (Supplementary Fig. 8). In addition, we measured the average weight of mice from each experimental group over 4 weeks from injection (Fig. 7b). We followed the mating capability and the number of pups delivered during six breeding rounds. No evident differences in behavior and development were observed between the off-springs from each experimental group during the first post-natal week. However, we observed that fertility was impaired in the females injected with cyclophosphamide and DPH (Fig. 7c, d). The mice treated with cyclophosphamide and DPH were infertile after three mating rounds, quite before those treated with cyclophosphamide. On the other hand, co-treatment of mice with GNF-2 and cyclophosphamide prolonged the fertility as compared to cyclophosphamide-treated mice and increased the cumulative number of pups compared to cyclophosphamide + DPH and cyclophosphamide groups (Fig. 7e). The gross morphology of the ovaries dissected from adult mice showed a clear difference in size between gonads of each experimental group. The analysis of ovarian sections by IHC assay with Msy2 confirmed the nearly complete absence of follicle reserve in the ovaries from cyclophosphamide + DPH and cyclophosphamide groups (Fig. 7f).

## Discussion

Cyclophosphamide is currently used for the treatment of pediatric cancers. A major concern is the ovarian reserve depletion induced by cyclophosphamide treatment. In the present study, we used a pre-pubertal mouse model to show in vivo the signaling axes induced by cyclophosphamide. We also tested small molecules that could limit the cyclophosphamide-induced toxicity by acting as putative “ferto-adjuvants“.

Genetically modified mouse models have facilitated the study of the signaling pathways induced in ovary by IR or chemotherapy. Gene targeting approaches may oversimplify the interpretation of the results. The lack of a single node/protein could affect signaling circuits at different levels and the observations reported here in knockout mice may be attributed to the multiple effects that are difficult to dissect. Small molecules offer unique advantages in terms of interpretation of results because of their transient inhibitory effects on targeted proteins (and/or pathways). Pharmaceutical inhibitors may have a direct relevance with patient care.

**Fig. 8:**
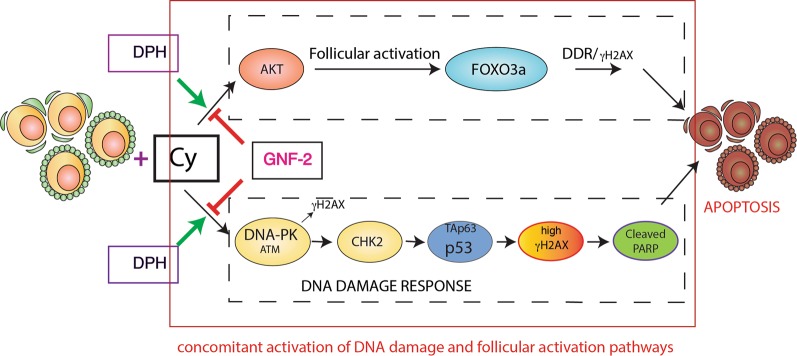
Proposal model. Concomitant administration of GNF-2 and cyclophosphamide results in the inhibition of the AKT-FOXO3a signaling axis and DDR, thereby preventing cyclophosphamide-induced apoptosis of oocyte reserve. On the contrary, DPH enhanced the activation of the AKT-FOXO3a signaling axis and DDR response following cyclophosphamide injection. Oocyte reserve was positive for both p-AKT/p-FOXO3a and p-ATM/γH2AX; thus, the presence of the early marker of DDR was consistent with the activation of the AKT-FOXO3 pathway. This observation suggests that oocytes leave their follicular quiescence to initiate the DNA damage signaling pathway that leads to oocyte apoptosis

Our study showed that cyclophosphamide-induced apoptosis in the ovarian reserve through signaling axes involving DNAPK/(ATM), CHK2, p53, and TAp63α. In addition, the co-treatment of cyclophosphamide and GNF-2 affected the DDR induced by cyclophosphamide in the ovary. We also showed that the reserve oocytes rescued from immediate degeneration were healthy enough to produce normal off-springs. Gross morphology and IHC analyses of ovary sections performed either before fertility test or after infertility detection in the first group of treated mice confirmed that the concomitant administration of GNF-2 and cyclophosphamide had long-term effects. Our data are reinforced through the use of an allosteric activator (DPH) that exerted opposite effects as compared to GNF-2. Co-treatment with DPH and cyclophosphamide enhanced the DDR induced by cyclophosphamide in ovaries and shortened mouse fertility. We also compared the effect of the transient administration of inhibitors targeting DDR apical kinases and observed mild protective effects exclusively at low doses of kinase inhibitors. Why allosteric compounds targeting ABL are more effective than ATP-competitive kinase inhibitors in rewiring apoptotic pathways induce by cyclophosphamide is questionable. We hypothesized that either (i) the theory of “burnout effect”^13^ or (ii) the direct DNA damage of follicle reserve in the ovary assaulted by cyclophosphamide may be associated with this effect. Both theories rely on the fact that the damaged somatic cells communicate with the oocyte. In this scenario, allosteric ABL compounds could affect follicle apoptosis by targeting the transmission of stress signals from pre-granulosa cells to the oocyte. Although the molecular details remain unclear, our current model suggests that ABL kinases may act as “allosteric devices” that contribute to fuel the molecular events underlying stress signaling/DDR in the follicles^27^. This model is supported by the observation that GNF-2 prevents the activation of the AKT-FOXO3 signaling axis induced by cyclophosphamide. We also found that DPH promotes the activation of the AKT-FOXO3a signaling axis following cyclophosphamide treatment and that the reserve oocytes were positive for both p-AKT/p-FOXO3a and p-ATM/γH2AX expression. Thus, the presence of the early marker of DDR is consistent with the activation of the AKT-FOXO3a pathway. These observations suggest that oocytes leave their follicular quiescence to initiate the DNA damage signaling pathways that end in oocyte apoptosis (Fig. 8). Whether this is a general mechanism occurring with other DNA-damaging chemotherapeutic drugs and IR is yet unknown.

Evidences suggest that ABL kinase inhibitors protect the ovarian reserve from cisplatin-induced degeneration^19–22^. However, the mechanism underlying the imatinib-mediated protection of oocytes remains debated. Genetic studies have shown that the oocyte ABL kinases are dispensable for cisplatin-induced TAp63α activation^10^. However, ABL kinases in complex with inhibitors may not recapitulate the results observed in ABL null mice, given that ABL proteins take part to opposing signaling pathways in cells^28^.

Recent studies have identified CHK2 and p53 as two important players in the efficient removal of oocytes with unrepaired meiotic DNA DBSs^8^. In addition, CHK2-dependent TAp63α phosphorylation is a key event induced in response to IR^6,8^. We show that cyclophosphamide-induced DNA damage checkpoint pathways that involve signaling of DNAPK to CHK2, which in turn communicates with p53 and TAp63, revising a previously proposed model^8^. We found p-p53 (Ser15) in the nucleus of the damaged oocytes, supporting the direct role of p53 in driving the apoptotic response of follicles. This observation is intriguing, as TAp63α is considered as a key player in quality control of germ cells. However, activated TAp63α exerts modest activity as a transcription factor as compared with p53, as we confirmed by ChIP experiments (Supplementary Fig. 1). Thus, the quality control and apoptotic response induced by cyclophosphamide require the predominant activity of p53.

A recent work showed that mouse models lacking PUMA (a pro-apoptotic protein) retained fertility after chemotherapy^29^. This observation supports the results that reserve oocytes from PUMA^−/−^ mice may sufficiently repair themselves to support healthy off-springs. These findings strengthen the argument that oocytes are in fact capable of efficient DNA repair in response to the inhibition of the apoptotic pathway in the ovary^30^.

The concomitant administration of GNF-2 and cyclophosphamide results in the inhibition of the AKT-FOXO3a signaling axis and DDR, thereby preventing cyclophosphamide-induced apoptosis of ovarian reserve. Thus, the feasibility of ferto-adjuvant therapies based on allosteric ABL compounds is established. With this in mind, we should consider that the systemic administration of allosteric inhibitors may interfere with the efficacy of cancer therapies. Clinical applications may warrant studies to improve the targeted delivery of these compounds to the gonads. Despite these efforts, the discovery of Asciminib (ABL001)^31,32^, the first allosteric ABL inhibitor used in clinic, offers promises to develop ferto-protective strategies suitable for pediatric cancer patients.

## Materials and methods

### Animals and injection

All procedures involving mice and care have been conducted at the Interdepartmental Service Centre- Station for Animal Technology (STA), University of Rome “Tor Vergata”, in accordance with the ethical standards, according to the Declaration of Helsinki, in compliance with our institutional animal care guidelines and following national and international directives (Italian Legislative Decree 26/2014, Directive 2010/62/EU of the European Parliament and of the Council). The ovaries were collected from CD-1 mice (Charles River) of 6 to 8 days old. Newborn mice (P6) were treated with intraperitoneal (ip) injection with PBS or cyclophosphamide (50, 75, 100, and 200 mg per kg of body weight). Mice were pre-treated with different inhibitors for 1 h before cyclophosphamide injection using a sterile micro syringe (Becton Dickson). Inhibitors used: GNF2 (range 15 mg–26 mg per kg of body weight), IMATINIB (12–42 mg per kg of body weight), NU (2–5 mg per kg of body weight), KU (10 mg per kg of body weight). Cyclophosphamide (BAXTER) was prepared fresh as concentrated 40 mg/ml in PBS. We dissolved Imatinib methane sulfonate salt (Novartis) in water, GNF-2 (SIGMA), NU (TOCRIS) and KU55993 (TOCRIS) in DMSO.

### Immunohistochemistry, Follicle counting, and statistical analysis

We prepared sections from ovaries fixed in MetaCarnoy solution (as previously described (Gonfloni et al 2009), embedded in paraffin and cut in slices of 5–7 μm of thickness. Sections were dewaxed, re-hydrated, and microwaved. Slices were then permeabilized with PBS triton 0,2 % and incubated with MSY-2 antibody (Santa Cruz). The staining was performed with immunocruz staining system for anti-goat antibody (Santa Cruz, sc-2023) and 3-aminoethyl-9-ethylcarbazole as substrate (AEC, Sigma). Sections were counterstained with hematoxylin and cover-slipped with Aquatex. Quantification of primordial and primary or secondary follicles was derived from histological analysis, counting Msy2-positive germ cells of mid-ovary sections. For each ovary (P9), several central slices (10 < *n* < 15) are included in the counting, with the exception of smaller peripheral slices (12–14 on average per each ovary). Quantification of primordial/primary follicle reserve is expressed as mean of immature follicles (primordial plus primary follicles) per single ovary. Average values for each ovary are represented as discrete points on a scatter plot. Mean value ± S.D. are shown in the scatter plot. The analysis of variance is evaluated with one-way ANOVA, with Turkey multiple comparison Test using PRISM 6 (Graph Pad software) (*P < 0.05; **P < 0.01; ***P < 0.001) or by unpaired Student’s *t* test where indicated.

### Immunofluorescence

We prepared sections from ovaries sections fixed in MetaCarnoy solution, embedded in paraffin and cut in slice of 5–7 μm of thickness. Sections were dewaxed re-hydrated and microwaved in sodium citrate 10 mM pH6, to expose the antigens. Unspecific-binding sites were blocked by incubating sections for 2 h in a blocking solution (PBS plus 1%glycine, 5% BSA, 5% FBS and 5% NGS (normal goat serum). Ovaries sections were then incubated overnight with antibodies against MSY-2, p63, p-ATM, γH2AX, p-DNA-PK, p-p53, p-CHK2, p-DNAPK, pAKT, p-FOXO3a and cleaved PARP. After washing in PBS triton 0,05%, tissue sections were incubated with Alexa 555-goat anti-mouse (life technologies) and alexa 488-goat anti rabbit (invitrogen).

### Immunoblot analysis

P7 dry ice–frozen ovaries were homogenized with a mini-pestle in ice-cold lysis buffer (50 mM Tris-HCl pH 7.5, 150 mM NaCl, 0.5% NP-40, 5 mM EDTA, 0.5% sodium deoxycholate, 1 mM phenylmethylsulfonyl fluoride, 1 mM sodium o-vanadate, 10 μg ml^−1^ Tosyl phenylalanyl chloromethyl ketone (TPCK), 10 μg ml^−1^, Tosyl-l-lysyl-chloromethane hydrochloride (TLCK) supplemented with protease inhibitors, all purchased from SIGMA). Equal amounts of protein extract (equivalent of one up to three ovaries) was loaded onto 6%, 8% or 12% SDS-PAGE gel and transferred to a nitrocellulose membrane (Amersham Bioscience).

### Tunel

Ovary sections were stained according to the Fluorescein In Situ Cell Death detection Kit (Roche Diagnostic) and analyzed with a fluorescent filter. We used the protocol recommended by the manufacturer. DAPI (Molecular Probes Inc.)

### RNA isolation an RT-qPCR

P7 dry ice–frozen ovaries were homogenized with a mini-pestle in TRIzol Reagent (Thermo Fisher Scientific), and RNA was extracted according to manufacturer’s instructions. To generate cDNA for RT-qPCR, total RNA was solubilized in ribonuclease-free water and used for reverse transcription by PrimeScript™ RT Reagent Kit (Perfect Real Time) (Takara). Primers used were obtained from Sigma-Aldrich and are as follows:

**Table:** 

Gene	Forward	Reverse
Puma	AGGGAAGGGAGGGCTGAAGG	GAGGCCAGGCCCAAAGTGAA
Noxa	CGCTGGTGCTGCCTACTGAA	GCCTTTCTCCCGGGCATCTC
GAPDH	AAGGGCTCATGACCACAGTC	CAGGGATGATGTTCTGGGCA

Real-time qPCR was performed using the SYBR® *Premix Ex Taq* (Tli RNase H Plus) (Takara) on a StepOne real-time PCR System (Applied Biosystems). All reactions were run as triplicates. Data were analyzed by the StepOne™ Software (v2.3) using the second-derivative maximum method. The fold changes in mRNA levels were relative to a control after normalization to GAPDH.

### Chromatin Immunoprecipitation (ChIP)

Ovaries collected were cross-linked for 15 min at room temperature with 1% formaldehyde. The reaction was stopped by 5 min incubation in 125 mM glycine. Chromatin was sonicated using Branson Sonifier, seven cycles for 20 s ON, 20 s OFF. Average size of sonicated DNA was around 500–1200 bp, as measured by agarose gel electrophoresis. Samples were pre-cleared with pre-adsorbed Salmon Sperm A-coupled Sepharose beads, and overnight immunoprecipitated with anti -P63 (CST 13109) or anti-p53 (DO-1 sc-126) antibodies. Normal mouse or rabbit IgG were used as controls. Precipitated chromatin complexes were eluted by 150 μl of TE-SDS buffer (1% SDS, 10 mM Tris-HCl, 5 mM EDTA) for 15 min. Finally, the protein-DNA cross-links were reversed by overnight incubation at 65 °C. DNA amplification was performed using SYBR® Premix Ex Taq (Tli RNase H Plus) (Takara) on a StepOne real-time PCR System (Applied Biosystems). All reactions were run as triplicates. Data were analyzed by the StepOne™ Software (v2.3) using the second- derivative maximum method. Results are expressed as fold enrichment respect to IgG control. Primers used are as follows:

**Table:** 

Gene	Forward	Reverse
Puma Response Element	CCTCTGGCTGCCGGGAAACCCCCC	CCGCCCCGCCTCTCGCTGGCTCC

### Reagents

Antibodies Msy-2 (sc-21316), Abl K-12 (sc-131), β-tubulin (sc-9104), p53 DO-1 (sc-126) and p-AKT (T308) (sc-16646-R) were purchased from Santa Cruz; antibody for p-H2AX (γH2AX) (05–636), H2AX (07–627) and p-Tyr (4G10) (05–321) were purchased from Millipore; antibody for p-DNA-PK (S2056) (SAB4504169) and P63 (Y4A3) (P3362) were purchased from SIGMA, antibody for p-ATM (S1981) (200-301-500) was purchased from Rockland, polyclonal antibody for p63 was an home-made rabbit serum; antibody for p-P53 (S15) (9284), p-CHK2 (T68) (2197), p-FOXO3a (S253) (9466), p-CHK2 (T68) (2197), P63 (CST 13109) and cleaved PARP (9544) were purchased from Cell Signaling Technology. Antibody for Abl (8E9) was purchased from BD-pharmingen. Secondary antibodies were purchased from Jackson Immunoresearch. All the antibodies were diluted in a blocking solution containing 5% BSA in PBS tween 0,05% for Western Blotting analysis and in a blocking solution containing 1% glycine, 5% FBS, 5% BSA and 5% NGS for immunofluorescence.

### Mating protocol

We injected five cohorts of newborn CD1 mice (25 total female pups) with a single dose of cyclophosphamide (100 mg per kg of body weight), GNF-2 (26 mg per kg body weight) and with cyclophosphamide in combination with GNF2 or DPH (20 mg per kg body weight). Injection with PBS was used as a control. Five-six weeks after injection, we mated the control and treated mice with proven fertile males at regular time intervals every 5–6 weeks. Once mating was established by the formation of the fertilization plug (this occurs within a week), we separated the females and allowed the pregnancies to progress until delivery. We kept the mice for a week with their pups and then separated them. After a week (without breast-feeding), we mated them again with proven fertile males.

## Supplementary information

Supplementary
