# Supplementary material for: Kinase-independent inhibition of cyclophosphamide-induced pathways protects the ovarian reserve and prolongs fertility
Source: Cell Death Dis. 2019 Sep 27;10(10):726. doi: 10.1038/s41419-019-1961-y (PMC6765024; doi:10.1038/s41419-019-1961-y)
Supplement: Supplementary file 1 — Supplementary [file 41419_2019_1961_MOESM1_ESM.docx]

**
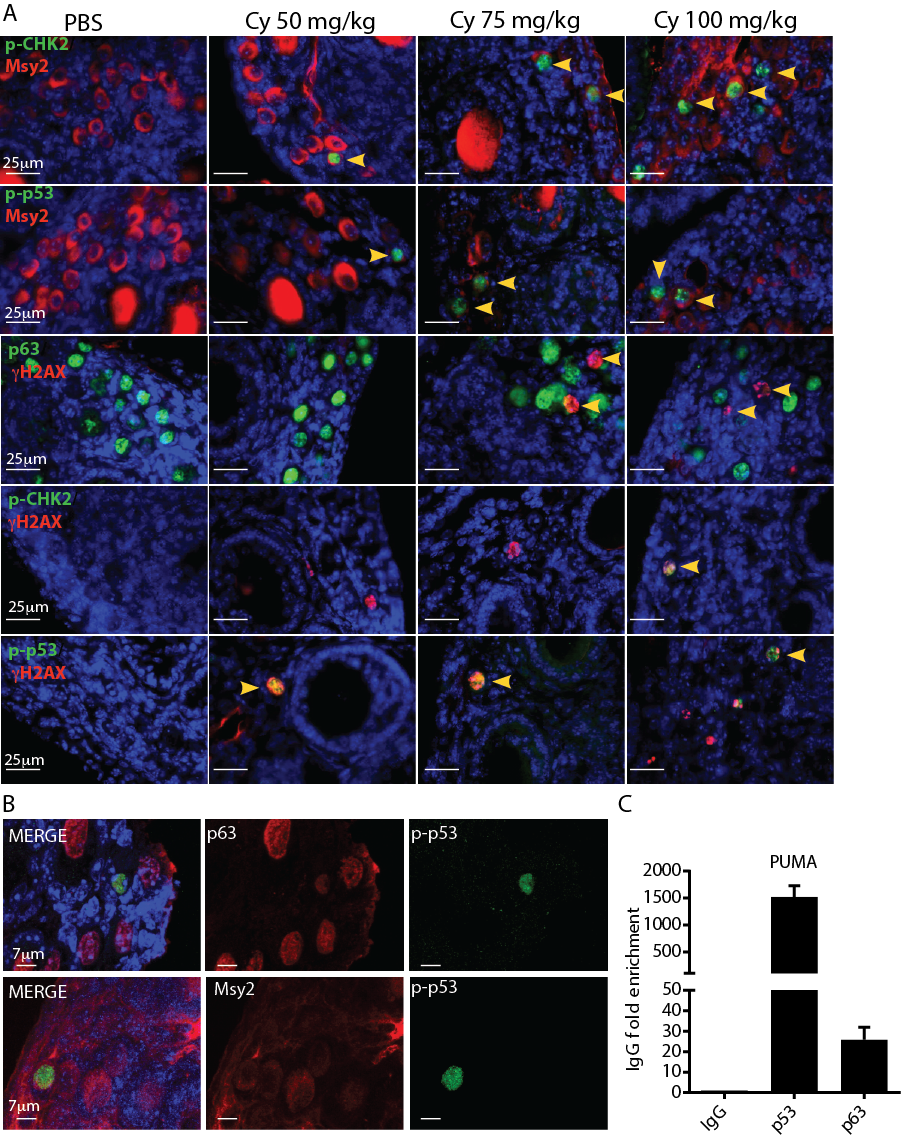
**

**Supplementary Fig.1** **Cy induces CHK2 and p53 activation in nucleus of reserve oocytes.**

P8 mice were injected with vehicle (PBS) or increasing concentrations of Cy (50, 75, 100 mg/kg) and sacrificed within 16 hrs from injection. (A) IF assay with two specific phospho-antibodies for CHK2, or p53 (green) and Msy2 (red), a cytoplasmic antigen of germ cells. Co-staining of p-CHK2 and γH2AX or p53 and γH2AX shows the activation of DNA damage response pathway in reserve oocytes*.* (B) Confocal images of middle ovarian sections from Cy-injected mice confirm the presence of pho-p53 in the nucleus of germ cells. Scale Bar magnification 25µm for IF assay and 7μm for confocal images. (C) ChIP analysis performed on P7 cyclophosphamide 100 mg/kg injected ovaries on Puma promoter regions with two specific antibodies for p53 or p63. Data are expressed as fold enrichment vs. background signal (IgG).


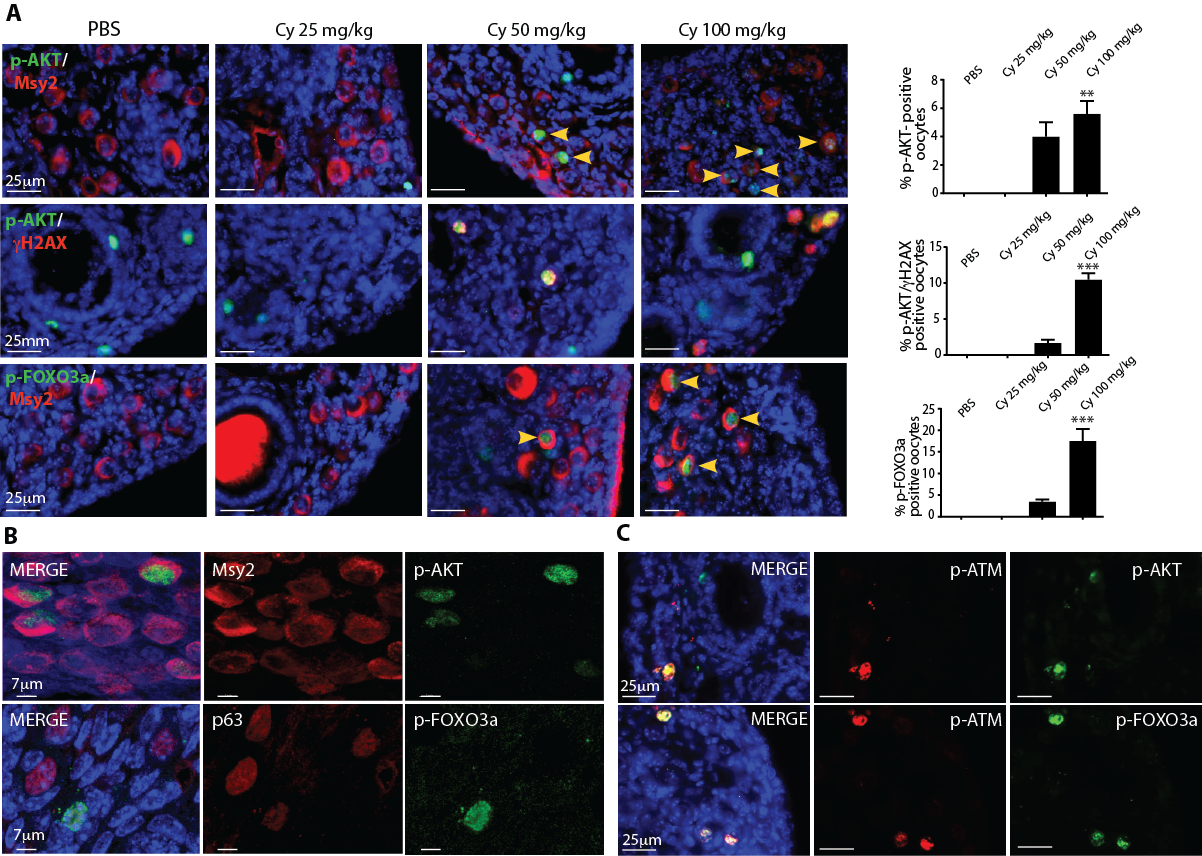


**Supplementary Fig. 2. Cy induces the AKT- FOXO3 pathway in the nucleus of the reserve oocyte.**

P7 mice were injected with vehicle (PBS) or increasing concentrations of Cy (25, 50 and 100 mg/kg) and sacrificed within 16 hrs from injection. AKT(T308) and FOXO3a(S253) phosphorylation are followed by IF assay with a phospho-specific antibody (green) and Msy2 (red), a cytoplasmic antigen of germ cells. Yellow arrows indicate oocytes positive for AKT (upper panel) and FOXO3a (lower panel). In the central panel, co-staining of p-AKT and γH2AX shows the activation of DNA damage response pathway in reserve oocytes. Quantification was obtained by counting several (6<x<8) middle ovarian sections derived from distinct ovaries. (B) Confocal images of middle ovarian sections from Cy-injected mice confirm the presence of p-AKT and p-FOXO3a in the nucleus of germ cells. (C) IF assay of middle ovarian sections from Cy-injected mice shows co-staining of p-ATM and p-AKT or pho-FOXO3a. Scale Bar magnification 25µm for IF assay and 7μm for confocal images. (A) Bar column represents mean ± s.d., statistical significance was determined using one-way analysis of variance (ANOVA) (**P<0.01; ***P<0.001 compared to PBS).


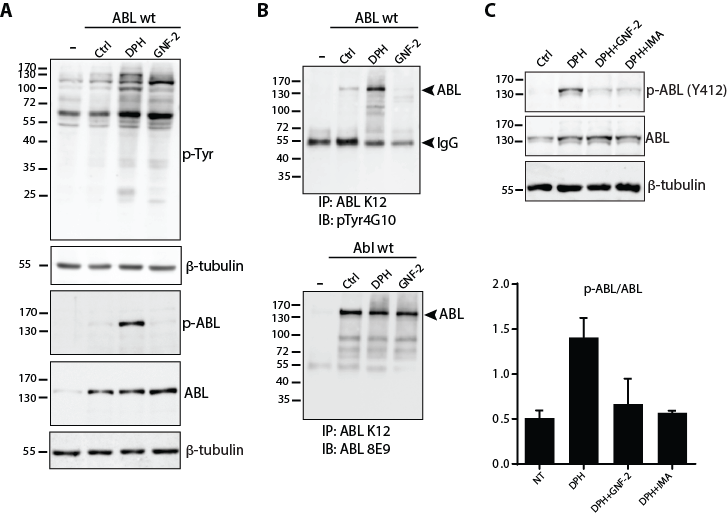


**Supplementary Fig.3 Allosteric compounds modulate ABL catalytic activity in H1299** Human lung carcinoma cell line H1299 was used for *in vitro* studies to monitor the ABL kinase activity following GNF-2, DPH and Imatinib exposure. (A) Western blotting of total lysate from H1299 cells transfected with a plasmid encoding wild type ABL. DPH treatment induces auto-phosphorylation of Y412 in the activation loop of ABL kinase, while GNF-2 has an opposite effect. (B) Immunoprecipitation assay with ABL polyclonal antibodies was done with the same H1299 extracts to further confirm the data. (C)Total lysates fromH1299 cells, treated with DPH and GNF-2 or Imatinib, show that the allosteric compounds affect the kinase activity of endogenous ABL.


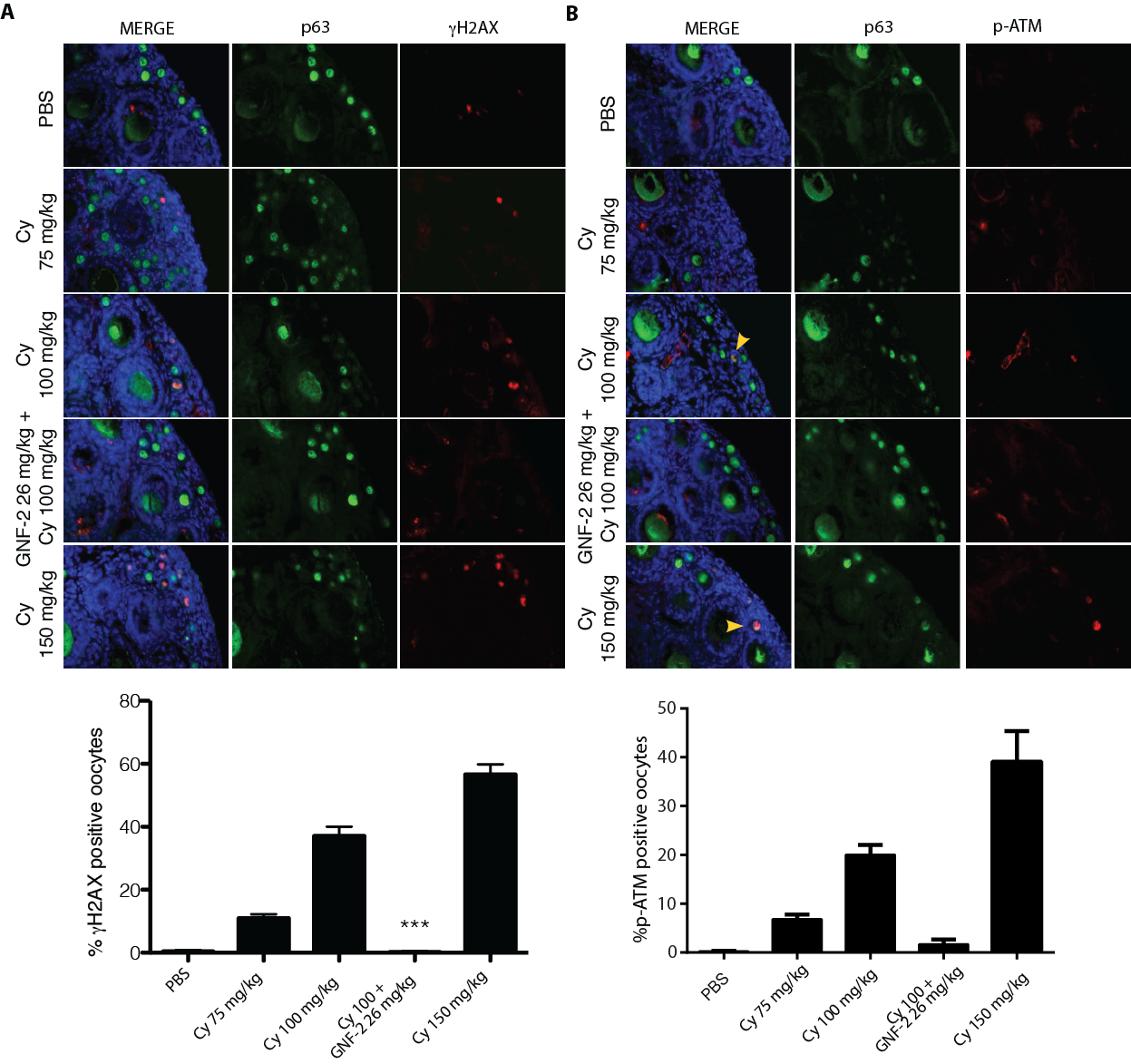


**Supplementary Fig.4 GNF-2 affects ATM phosphorylation in the follicle reserve**

P8 mice were injected with vehicle (PBS) or increasing concentration of Cy (75, 100 and 150 mg/kg). Cy 100 mg/kg was injected alone or in presence of GNF-2 26 mg/kg. Co-staining of p63 (green) and γH2AX or phosphorylated ATM (red) shows an increase in the number of oocytes positive to γH2AX as indicated in the graph (A). Oocytes with a reduced level of TAp63 show a high expression of phosphorylated ATM. (B) Co-treatment with GNF-2 prevents both ATM activation and γH2AX in the reserve oocytes. Quantification was obtained by counting several (6<x<8) middle ovarian sections per condition. Quantification of the number of positive oocytes is expressed as the average of follicle number per ovarian section ± s.d. Statistical significance was determined using one-way analysis of variance (ANOVA) (**P<0.01; ***P<0.001 compared to Cy 100 mg/kg). Scale Bar magnification 25µm.


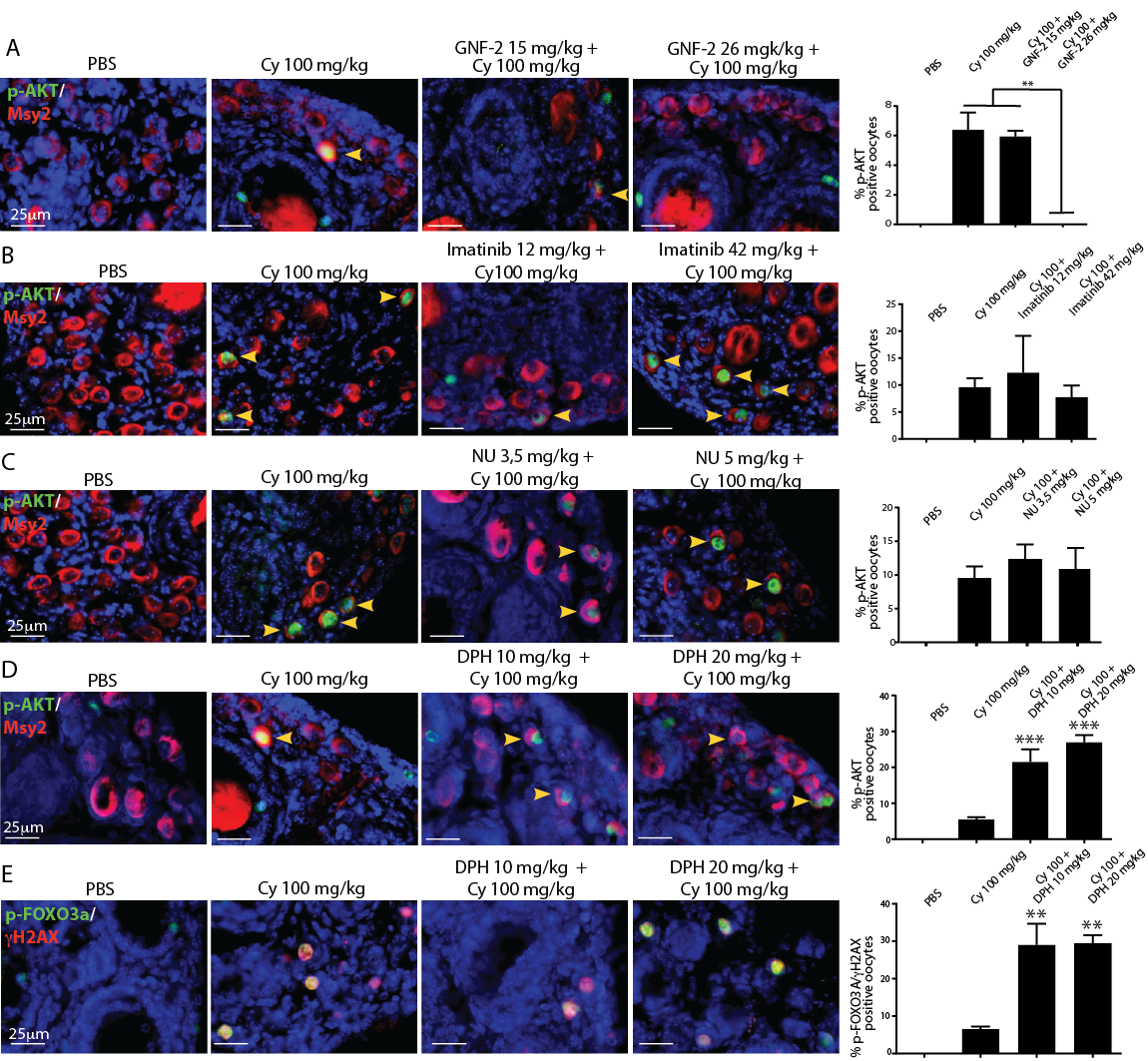


**Supplementary Fig.5 Allosteric compounds modulate AKT-FOXO3 signaling axis induced by Cy in the nucleus of reserve oocytes.**

P7 mice were injected with vehicle (PBS) or Cy (100 mg/kg) with/out increasing concentration of GNF-2 (A), Imatinib (B), NU7441 (C) or DPH (D) and were sacrificed within 16-24 hrs from injection. Ovarian sections were analysed by IF assay with specific phospho-antibodies for AKT (T308) (green), FOXO3a(S253) (green), Msy2 (red), or γH2AX (S139). (A) GNF-2 (26 mg/kg) co-treatment prevents the phosphorylation of AKT in the nucleus of reserve oocytes; (B, C) While, Imatinib or NU7441 (DNAPK inhibitor) do not prevent AKT phosphorylation induced by Cy injection; D) DPH co-treatment enhances the phosphorylation of AKT and well as the phosphorylation of FOXO3a. Quantification of the p-AKT positive cells was obtained by counting several (6<x<10) middle ovarian sections derived from distinct ovaries. Bar column represents mean ± s.d., statistical significance was determined using one-way analysis of variance (ANOVA) (**P<0.01; ***P<0.001 compared to Cy 100 mg/kg). Scale Bar magnification 25µm.


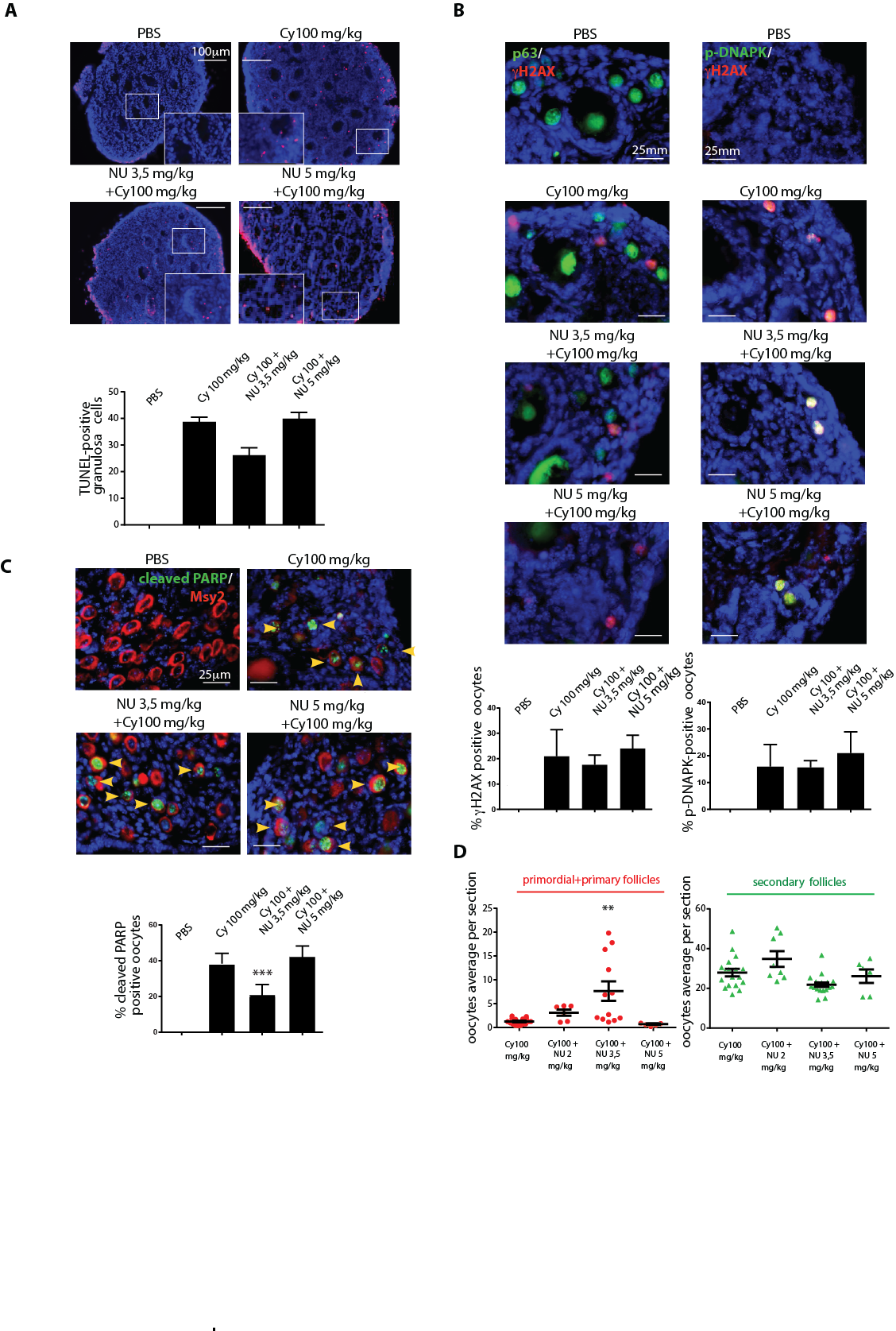


# **Supplementary Fig.6 NU7441 does not prevent oocytes loss induced by Cy**

P7 mice were injected with vehicle (PBS) or Cy (100 mg/kg) with/out increasing concentration of NU7441 (2 mg/kg, 3.5 mg/kg and 5.3 mg/kg) and were sacrificed within 16-24 hrs from injection. (A) Ovarian sections were analysed by in situ TdT-mediated dUTP nick-end labelling (TUNEL). The graph shows the quantification of TUNEL-positive cells. Quantification of TUNEL-positive cells was obtained by counting six different middle ovarian sections derived from three distinct ovaries. (B) γH2AX and DNAPK activation is followed by IF assay with phospho-specific antibodies, p63 was used as nuclear marker for germ cells. Quantification was obtained by counting several (6<x<8) middle ovarian sections derived from three distinct ovaries. Co-staining of p-DNAPK and γH2AX shows the activation of DNA damage response in reserve oocytes. Quantification was obtained by counting several (6<x<8) middle ovarian sections derived from three distinct ovaries. (C) Ovarian reserve apoptosis was assessed by IF assay with two specific antibodies cleaved PARP (green) and Msy2 (red), a cytoplasmic antigen of germ cells. Quantification of cleaved PARP positive cells was obtained by counting several (6<x<8) middle ovarian sections derived from three distinct ovaries. Bar column represents mean ± s.d., statistical significance was determined using one-way analysis of variance (ANOVA) (***P<0.001 compared to Cy 100 mg/kg) (D) Ovaries collected three days after injection were analysed by IHC assay with Msy2 antibody (see Fig.5 B). Ovaries of three independent experiments were analysed, each dot in the box plot represents the average numbers of follicles (Primordial/primary and secondary) per section of each gonad collected. Statistical significance was determined using one-way analysis of variance (ANOVA) (***P*<0.01 ****P*<0.001 compared to Cy 100 mg/kg). Scale Bar magnification 100µ for TUNEL assay and 25 µ for IF assay.


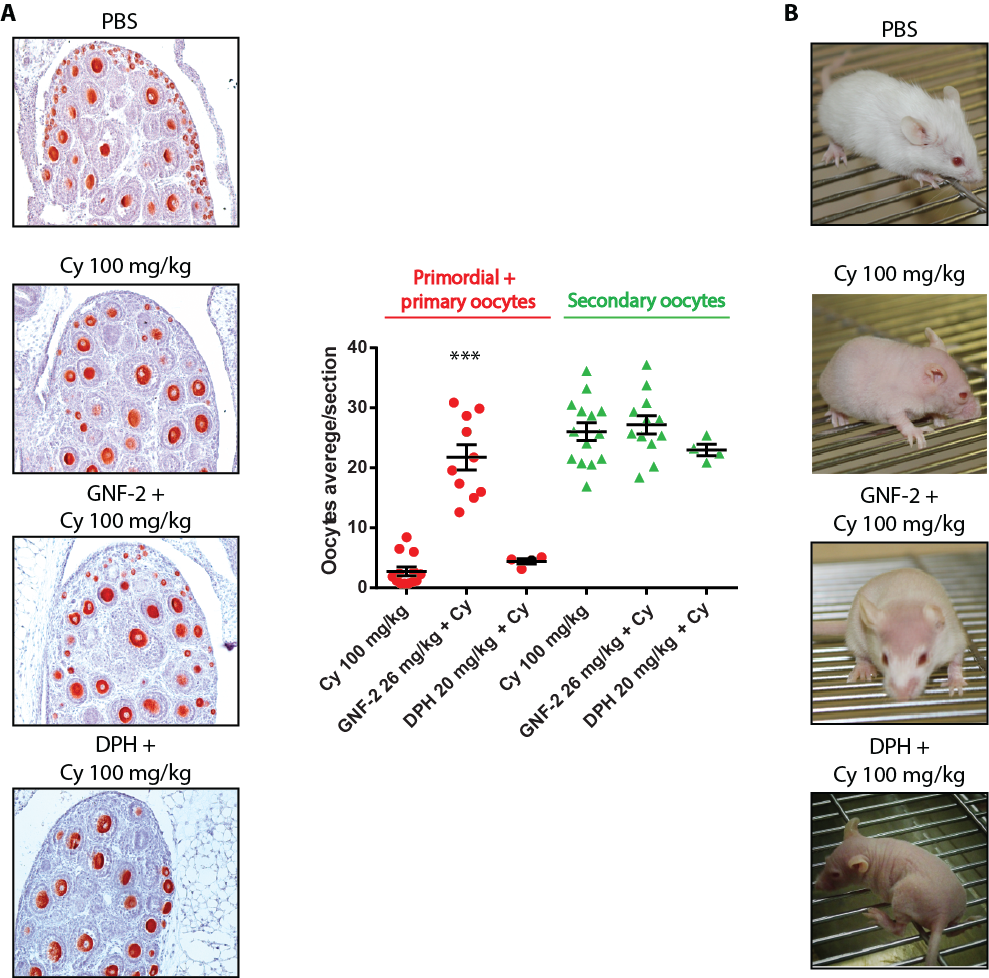


**Supplementary Fig.7. Effects of concomitant administration of allosteric ABL compounds in Cy-treated mice**

(A) Ovaries of each experimental group were dissected three days after injection and analysed by IHC assay with Msy-2 antibody. Several ovaries from independent experiments were analysed, each dot in the box plot represents the average numbers (Primordial/Primary, or Secondary) follicles per section of each gonad collected. Statistical significance was determined using one-way analysis of variance (ANOVA) (***P<0.001 compared to Cy 100 mg/kg). Scale Bar magnification 100µm. (B) Mice photos were taken about two weeks after injection.


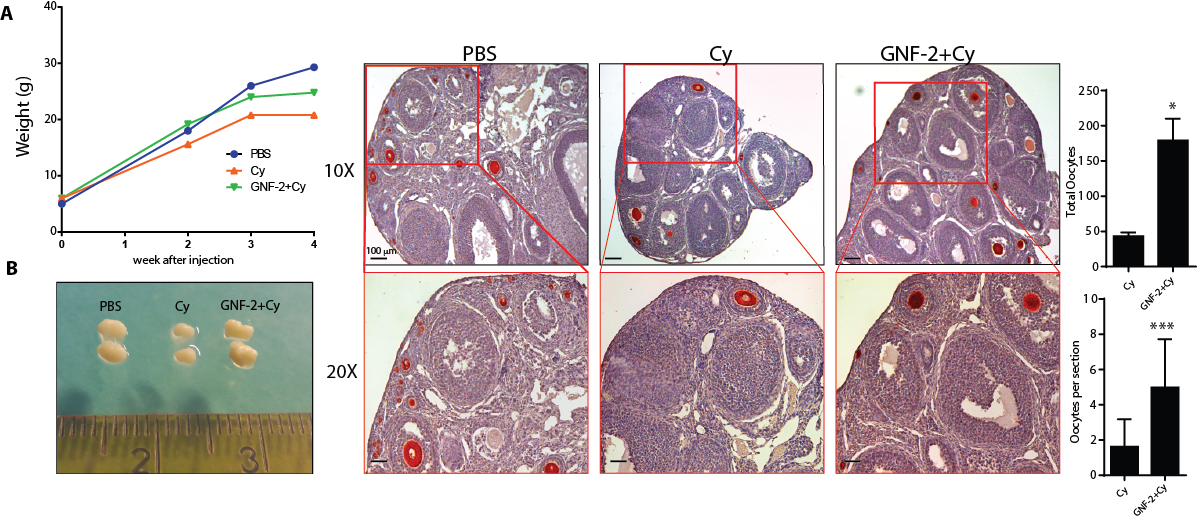


**Supplementary Fig.8. Long-term protection of GNF-2 is evaluated in pubertal ovaries**

(A) Graph of average weight following 4 weeks from injection for each experimental group. (B) Gross morphologies of ovaries dissected from pubertal adult mice before the fertility test. (C) Representative sections of the same ovaries showed in (B) were analyzed by IHC assay with Msy2. Quantification of follicle reserve is also shown. Scale bar magnification 100μm. The data are expressed as mean ± s.d. (n=4). Statistical significance was determined by unpaired Student’s *t* test (*P<0.05; ***P<0.001 compared to Cy 100 mg/kg).
